# Prevalence of Group B Streptococcus Recto-Vaginal Colonization, Vertical Transmission, and Antibiotic Susceptibility Among Pregnant Women in Ethiopia: A Systematic Review and Meta-Analysis

**DOI:** 10.3389/fpubh.2022.851434

**Published:** 2022-05-16

**Authors:** Habtamu Bekele, Adera Debella, Tamirat Getachew, Bikila Balis, Dawit Tamiru, Addis Eyeberu, Getahun Tiruye, Mohammed Abdurke Kure, Sisay Habte, Bajrond Eshetu, Lemma Demissie Regassa, Sinetibeb Mesfin, Adisu Alemu, Yadeta Dessie, Kasiye Shiferaw

**Affiliations:** ^1^School of Nursing and Midwifery, College of Health and Medical Sciences, Haramaya University, Harar, Ethiopia; ^2^School of Public Health, College of Health and Medical Sciences, Haramaya University, Harar, Ethiopia

**Keywords:** prevalence, recto-vaginal, pregnant women, GBS, colonization, antibiotic susceptibility

## Abstract

**Background:**

Maternal Group B Streptococcus (GBS) recto-vaginal colonization is the most common route for early onset neonatal GBS diseases. A good understanding of the rate of maternal GBS colonization, vertical transmission rate, and antibiotic susceptibility profiles is needed to formulate a broad protection mechanism, like vaccine preparation. For that reason, this meta-analysis aimed at determining the pooled prevalence of GBS recto-vaginal colonization, vertical transmission rate, and antibiotic susceptibility profiles in Ethiopia.

**Methods:**

Both published and unpublished studies were searched from MEDLINE/PubMed, CINAHL (EBSCO), Embase, Cochrane Library, SCOPUS, Web of Sciences databases, and Google Scholar. Independent selection was then carried out by the authors based on the eligibility criteria and data extraction using Microsoft excel. The authors then used STATA version 14.1 software for further cleaning and analysis. The review was based on the Preferred Reporting Items for Systematic Reviews and Meta-Analyses) PRISMA guidelines. Using the random-effect model, the prevalence with a 95% confidence interval (CI) and forest plot were used to present the findings. Besides, the studies' heterogeneity was assessed using Cochrane chi-square (I^2^) statistics, while Egger intercept was used to assess publication bias.

**Results:**

This review included nineteen studies. The pooled prevalence of recto-vaginal colonization was 15% (95% CI: 11, 19), while the prevalence of vertical transmission was 51% (95% CI: 45, 58) and highest-level susceptibility to vancomycin was 99% (95% CI: 98, 100). However, the GBS susceptibility to tetracycline was 23% (95% CI: 9, 36).

**Conclusions:**

Nearly one out of seven pregnant women in Ethiopia had recto-vaginal colonization of GBS. As a result, half of the pregnancies end with vertical transmission of GBS. Hence, the review emphasizes that policy and programs should consider planning and implementing prophylactic programs.

**Systematic Review Registration:**

https://www.crd.york.ac.uk/prospero/display_record.php?ID=CRD42021287540.

## Introduction

Group B streptococcus (GBS) is a member of gram-positive streptococci that causes an invasive fetal infection in newborn (1). It can be early-onset disease (EOD) and late-onset disease (LOD) if it occurs in the first week of delivery and from week 1–3 months after delivery, respectively (2–4). About 15–40% of healthy women's colon and vagina have GBS. Evidence pointed out that urinary tract infection (UTI), endometritis, chorioamnionitis, sepsis, and meningitis are associated with maternal colonization of GBS (2, 4–9). Moreover, pregnant women may encounter premature rupture of membranes (PROM), stillbirths, and low birth weight (LBW) babies due to GBS vertical transmission (10–12).

Vertical transmission of GBS occurs during pregnancy or the birth process from the genitourinary or gastrointestinal tract of colonized pregnant women (13). Mortality risk among newborns with GBS colonization is 6.6-fold higher (14). There were 48 infants with early-onset group B streptococcus (EOGBS), which is 1.4 per 1,000 neonates in the general population and 7.8 per 1,000 in women with GBS (15). Neonates with EOGBS disease are more likely to have respiratory distress disease and convulsions and require longer hospital stays (16). Case fatality is highest in Early onset of Neonatal Death when it occurs within 24 h of birth (12). Moreover, survivors suffer from uncontrolled seizures, impaired psychomotor development, profound intellectual disability, blindness, and deafness (17).

Globally, the prevalence of vaginal colonization by GBS among pregnant women varies across countries (18). For instance, its magnitudes ranged from 9.1 to 26.7% in Iran (8), and 8 to 15% across Europe (9). The prevalence of GBS in African countries, such as Egypt, Malawi, Nigeria, and Tanzania, ranges from 11.3 to 23% (19–22). Several studies across regions in Ethiopia revealed a varied proportion of recto-vaginal colonization of GBS among pregnant women (i.e., 7.2 to 20.9%) (18, 23–25). Worldwide, vertical transmission of GBS shows considerable variation within and between geographic regions, ranging from 11.2 to 57.7% (26, 27).

A cross-sectional study conducted by Woubshet et al. pointed out that the majority of the isolates were susceptible to ampicillin (95.5%) and penicillin G (90.9%). However, half of the isolates were resistant to erythromycin and clindamycin. Despite the fact that some GBS isolates are resistant to gentamicin, it is used in conjunction with penicillin to treat severe GBS-associated infections (28). Similarly, studies conducted in Nigeria (35.3%) (29) and Palestine (43%) agree with these findings (30). Countries that introduced prevention strategies, including universal routine antenatal GBS screening, identification of risk factors, and offering antibiotic prophylaxis at delivery, were able to significantly reduce the disease (22, 31). However, most of the sub-Saharan African countries do not have clear guidelines for prevention, despite the high rate of vertical transmission (27, 32, 33).

Despite few studies having been conducted on recto-vaginal colonization of GBS, its vertical transmission, and antimicrobial susceptibility among pregnant women in Ethiopia, there is, however, no national-level data or cumulative evidence to show the exact extent of the wider impact. The prevalence of maternal colonization with GBS and antibiotic susceptibility profiles in the Ethiopian context has been investigated on a small scale and in a fragmented fashion. Hence, it is imperative to conduct a comprehensive study that can aggregate the previous studies and make the findings available to policy- and decision-makers. Therefore, this systematic review and meta-analysis are conducted to generate national and reliably pooled-evidence on recto-vaginal colonization of GBS, its vertical transmission, and antimicrobial susceptibility among pregnant women in Ethiopia.

## Methods

### Protocol and Registration

This review was based on the Preferred Reporting Items for Systematic Reviews and Meta-Analyses (PRISMA) guidelines (34). The review was registered by the International prospective register of systematic reviews with identification CRD42021287540.

### Information Sources and Search Strategy

Both published and unpublished studies were searched from MEDLINE/PubMed, CINAHL (EBSCO), Embase, Cochrane Library, SCOPUS, Web of Sciences databases, and Google Scholar by using a combination of Boolean logic operators (AND, OR, NOT), Medical Subject Headings (MeSH) as well as keywords. An additional search was also conducted using Google scholar and Mednar. The search strategy includes: “Recto-vaginal colonization” [Title/Abstract] or “Vertical transmissions'“[Title/Abstract] or “Antimicrobial susceptibility””[Title/Abstract] ‘AND “Ethiopia” [MeSH Terms]), (“Recto-vaginal colonization, vertical transmission and antimicrobial susceptibility” [exactly on title] AND “Ethiopia” [any field]), (“Recto-vaginal colonization, vertical transmission and antimicrobial susceptibility ”[all field] AND “Ethiopia” [all filed]) (Supplementary File 1). In addition, the investigators searched manually for gray literature and other relevant data sources such as unpublished thesis and articles with planned dates of coverage.

### Eligibility Criteria

All observational studies conducted in Ethiopia, which reported the prevalence of recto-vaginal colonization, and/or vertical transmission, and/or antimicrobial susceptibility of GBS among pregnant women, were eligible. The eligibility criteria also included English language articles and facility-based studies conducted from 12 December 2012 to October 2021. Articles, theses, and articles fulfilling the aforementioned inclusion criteria were considered for the systematic review and meta-analysis.

### Study Selection

**Figure 1:**
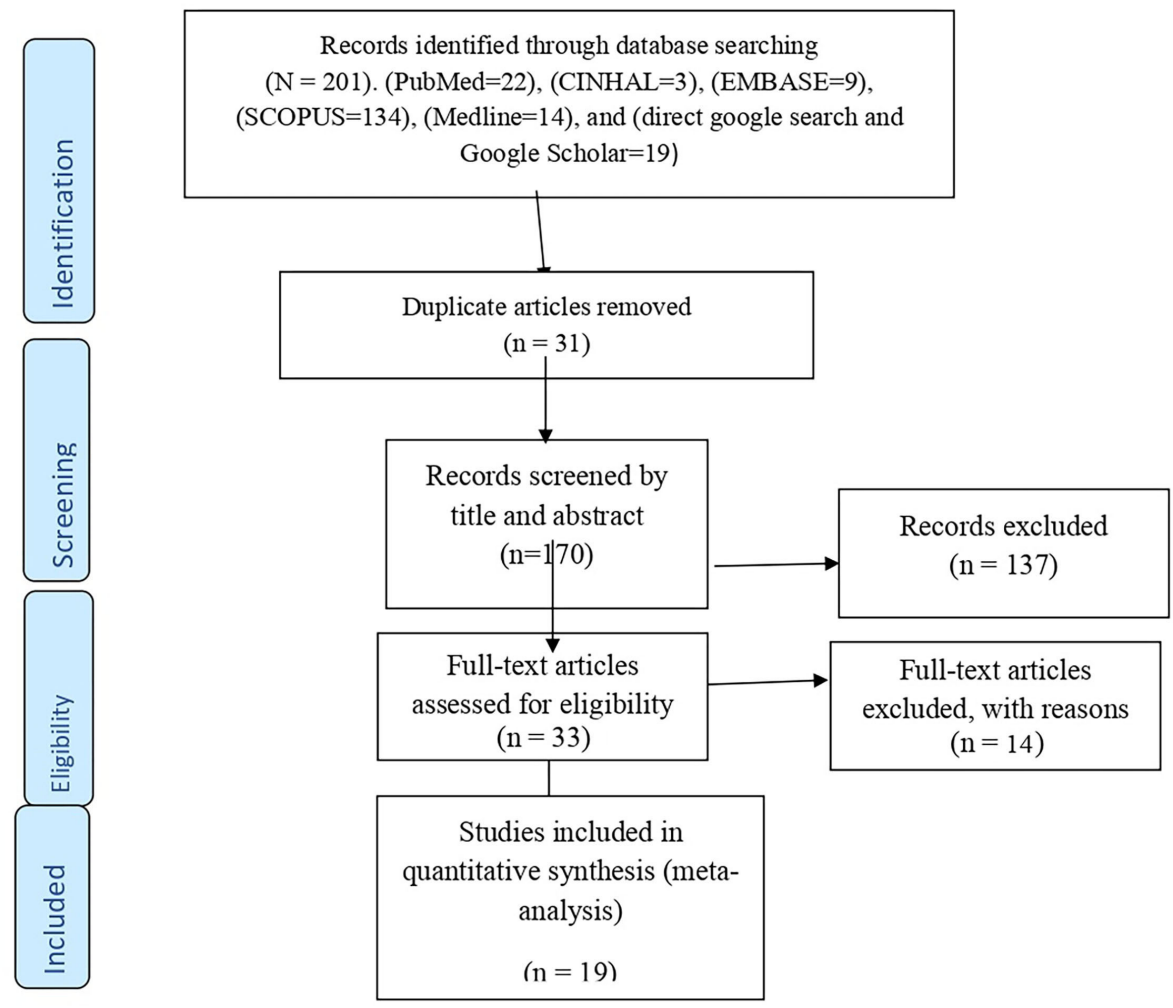
PRISMA flow diagram showing the selection process of eligible articles for systematic review and meta-analysis.

All the searched and found articles were exported to the EndNote X8 citation manager, and duplicates were deleted. These were then screened carefully by reading the titles and abstracts. The review authors (AE, TG, AD, and MA) independently screened the titles and abstracts of the identified articles using the eligibility criteria. Full-text articles were further evaluated for eligibility, as needed. Any disagreement among the authors was resolved *via* discussion and critical reflection. The overall study selection process is presented using the PRISMA statement flow diagram (35) (Figure 1).

### Data Extraction

The review authors (AE, TG, AD, and MA) independently extracted the data. A pre-defined Microsoft excel 2016 format was used to extract the data from the included articles using author(s), publication year, region of study, study setting, study design, sample size, data collection technique, and the primary outcome of interest (Table 1). The accuracy of the data extraction was verified by comparing the results of the independently extracted data.

**Table 1:** Characteristics of studies revealing recto-vaginal colonization of Group B streptococcus Ethiopia, 2021.

**References**	**Year**	**Study area**	**Region**	**Design**	**RVC**			**VT**			**Factor associated with RVC of GBS**
					**Sample size**	**Event**	**%**	**Sample size**	**Event**	**%**	
Ali et al. (36)	2020	Adama	Oromia	cross-sectional	280	37	13.20	37	21	56.7	
Woldu et al. (23)	2014	Addis Ababa	Addis Ababa	cross-sectional	300	22	7.20				
Shiferaw et al. (37)	2019	Arbaminch	SNNP	cross-sectional	281	24	8.50				
Randis et al. (38)	2018	Harar	Harari	cross-sectional	1688	231	13.68				
Ali et al. (39)	2019	Hawassa	Sidama	cross-sectional	280	44	15.70	44	26	59.1	
Mengist et al. (40)	2016	Jimma	Oromia	cross-sectional	126	31	24.60				
Vielot et al. (41)	2020	Jimma	Oromia	cross-sectional	135	22	16.30				**-**Preterm delivery (AOR: 6.3 (1.42, 28.3), -UTI (AOR:6.4, (1.95. 21.1)
Ali et al. (42)	2020	Addis Ababa	Addis Ababa	cross-sectional	280	65	23.20	65	32	49.2	
Giachew et al. (43)	2019	Gondar	Amhara	cross-sectional	385	98	25.50				- Meconium stained amniotic fluid (AOR 3.01: 95% CI (1.22, 7.43). -Prolonged PROM (AOR: 1.89, 95%CI (1.01, 3.41).
Schonfeld et al. (44)	2017	Asella	Oromia	longitudinal study	580	14	2.40				
Mohamed et al. (45)	2012	Hawassa	Sidama	cross-sectional	139	29	20.90				
Mengist et al. (46)	2017	Nekemte	Oromia	cross-sectional	245	22	8.90				GA, > 37wk (AOR: 2.1(1.2, 11.6). Married, (AOR:3.2, (1.8, 11.6)
Gebremeskel et al. (24)	2015	Adigrat	Tigrai	cross-sectional	150	17	11.30				
Fantahun et al. (47)	2020	Addis Ababa	Addis Ababa	cross-sectional	250	59	23.60	250	28	11.2	
Alemseged et al. (25)	2015	Mekele	Tigrai	cross-sectional	139	19	13.70				
Assefa et al. (48)	2018	Addis Ababa	Addis Ababa	Cross sectional	281	41	14.60				
Gizachew et al. (49)	2020	Gondor	Amhara	Cross sectional	385	98	25.50	98	62	63.2	
Yadeta et al. (50)	2018	Harar	Harari	Case control	**-**	**-**	**-**	231	104	45.05	
Leykun et al. (51)	2021	Bahirdar	Amhara	Cross-sectional	292	54	18.5	54	22	40.7	Preterm delivery (AOR: 2.77, 95% CI (1.14, 6.68). History of stillbirth (AOR:3.13, 95% CI (1.13, 8.70)

*GBS, group B streptococci; PROM, Premature rupture of Membrane; RVC, recto-vaginal colonization; VT, vertical transmission; UTI, urinary tract infection; AOR, adjusted odds ratio*.

### Data Item

The major outcome of interest of this review was the pooled proportion of GBS colonization of pregnant women, vertical transmission of GBS, and antibiotic resistance profiles reported from different studies across Ethiopia. Sub-group analysis was done based on Ethiopia's current political-geographic regions. Measurement for recto-vaginal colonization was made using swabs collected and inoculated into 1 ml Todd Hewitt Broth supplemented with gentamicin and nalidixic acid to avoid the growth of contaminants. After 18–24 h of incubation, all broths were sub-cultured on 5% sheep blood agar for isolation of GBS. The proportion of susceptibility of GBS to the 9 different antibiotics was calculated by dividing the numbers of susceptible isolates by the total number of GBS isolated from pregnant women.

### Risk of Bias

The authors critically evaluated the risk of bias from individual studies using the Joanna Briggs Institute Quality Assessment Tool for observational studies. To minimize the risk of bias, a comprehensive search (electronic/database search and manual search) for published, unpublished, institutional, or community-based studies was carried. Cooperative work among the authors was also considered seriously to reduce bias. All worked together in setting a schedule for the selection of articles based on the clear objectives and eligibility criteria, in deciding the quality of the articles, in regularly evaluating the review process, and also extracting and compiling the data.

### Critical Appraisal of the Studies

The methodological reputability and quality of the findings of the included studies were critically evaluated using the quality assessment tool for observational studies (cross-sectional, case-control, and cohort studies) developed by the Joanna Briggs Institute (JBI) (52) (Supplementary File 3). The two groups of the authors, Group 1 (AD and MA) and Group 2 (AE, KS, and TG), independently evaluated the quality of the studies. The mean score of the two groups was taken for a final decision. The differences in the inclusion of the studies were resolved by consensus. The included studies were evaluated against each indicator of the tool and categorized as high-, moderate-, and low-quality. A high-quality score is above 80%, moderate-quality is 60–80%, and low-quality is below 60%. Studies with a score greater than or equal to 60% were included. This critical appraisal was conducted in order to assess the internal validity (systematic error) and external validity (generalizability) of the studies and to reduce the risk of biases.

### Statistical Analysis

Data synthesis and statistical analysis were conducted using STATA version 14.1 software. The random-effect model of analysis was adopted as a method of meta-analysis because it reduced the heterogeneity of included studies. A meta-analysis of observational studies was carried out based on the recommendations of the I^2^ statistic described by Higgins et al. (I^2^ >75/100% suggesting considerable heterogeneity). The *p*-value of < 0.05 for I^2^ statistics was used to determine the presence of heterogeneity. Similarly, low, moderate, and high heterogeneity were assigned to I^2^ test statistics results of 25, 50, and 75%, respectively. The results were reported using the pooled prevalence with a 95% confidence interval (CI) and forest plot.

Also, sub-group analyses were conducted using publication years and regions in Ethiopia to identify the sources of heterogeneity among the studies. The investigators checked for potential publication bias using visual inspection of a funnel plot (53) and Egger's regression test. Publication bias was assumed for *p* > 0.10. The results of the review were reported according to the PRISMA guidelines. The findings of the included studies were first presented using a narrative synthesis and secondly by a meta-analysis chart.

## Results

### Search Results

A total of 201 article were identified through the electronic databases and other relevant sources. From all identified studies, 31 articles were removed due to duplication while 170 studies were reserved for further screening. Of these, 137 were excluded after being screened according to titles and abstracts. Of the 33 remaining articles, 14 studies were excluded due to studies did not present the outcome of interest and other relevant issues related to outcome variables. Finally, 19 studies that fulfilled the eligibility criteria were included for the systematic review and meta-analysis.

### Study Characteristics

The review included studies from regions in Ethiopia, though the majority of the studies were from Addis Ababa and Oromia. Eighteen cross-sectional and one case-control studies (i.e., reported magnitude and total at-risk population) were included in the meta-analysis (23–25, 36, 37, 39, 40, 42–51, 54, 55). The sample size of the included studies ranged from 126 to 1,688. A total of 5,924 study samples were included, among which 927 pregnant women were with recto-vaginal colonization of GBS (Table 1).

### The Pooled Effect Size of Recto-Vaginal Colonization of GBS

**Figure 2:**
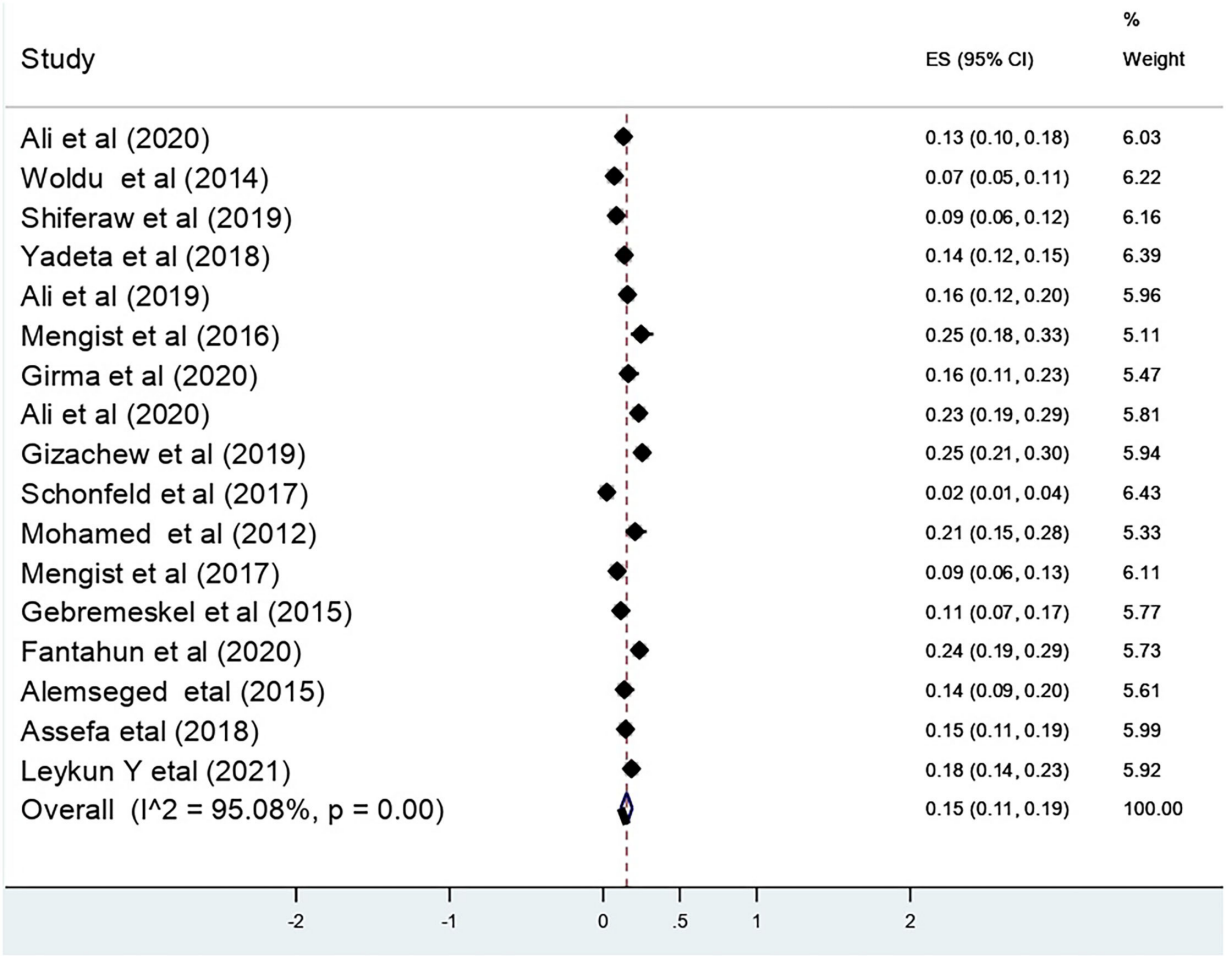
Recto vaginal colonization of GBS among pregnant women in Ethiopia 2022.

**Figure 3:**
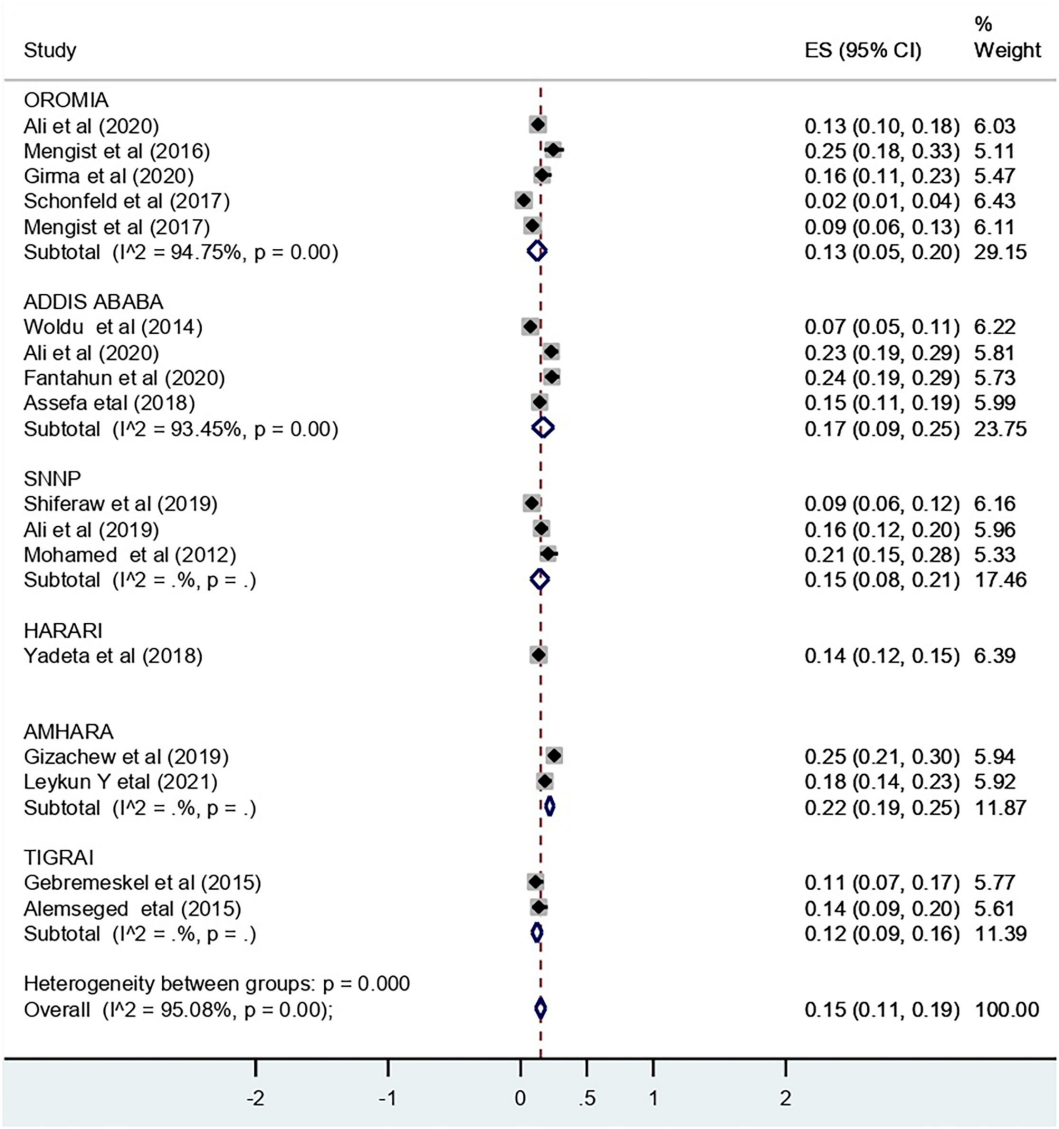
Subgroup analysis by region for the pooled prevalence of RVC of GBS among pregnant women in Ethiopia, 2022.

The pooled effect size for recto-vaginal colonization of GBS was 15% (95% CI: 11, 19) (Figure 2). Sub-group analysis showed that the highest pooled prevalence was from Addis Ababa city, i.e., 17% (95% CI: 9, 25) (Figure 3).

### Heterogeneity of the Studies

The overall substantial heterogeneity across the studies on recto-vaginal colonization of GBS was I^2^ = 95.08%, *p* < 0.001. Sensitivity analysis was performed for the outcome variable to observe a significant change in risk ratio and CI. The meta-analysis result showed that there was no substantial difference in the overall risk ratio during the sequential removal of each study from the analysis.

### Publication Bias

**Figure 4:**
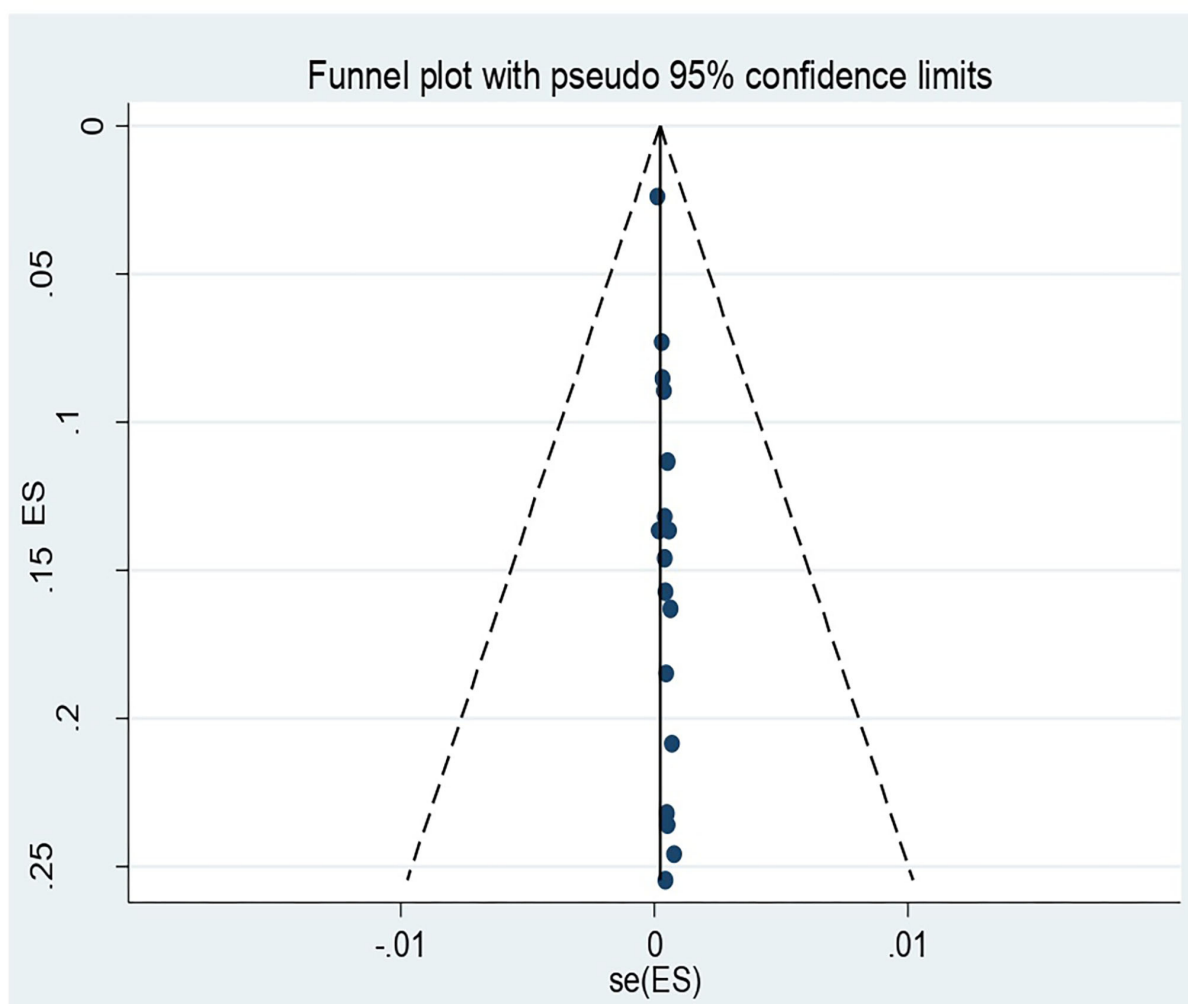
Funnel plot for recto vaginal colonization of Group B streptococcus among pregnant women 2022.

The Egger's regression test revealed evidence of publication bias among the included studies (*p*-value = 0.001). Nevertheless, a visual inspection of the funnel plot revealed symmetry (Figure 4).

### Meta-Regression to Check the Heterogeneity

A meta-regression analysis was conducted with assumptions of statistically significant heterogeneity of I-square test statistics <0.05. However, the meta-regression analysis found no significant variable to indicate there was any heterogeneity. What this means is that there was no statistically significant level covariation among the sample size, as well as across the publication years of the included studies. Therefore, if significantly present, heterogeneity might be explained by other factors not included in this review (Table 2).

**Table 2:** Results of meta-regression analysis to check heterogeneity on rectovaginal colonization of GBS among pregnant women in Ethiopia, 2021.

**Variables**	**Coefficients**	**SE**	**P**	**[95% Conf. Interval]**
Publication year	0.0162422	0.023458	0.499	−0.0337526, 0.066237
Sample size	6.174027	1.49259	0.001	2.992646, 9.355407

### The Pooled Effect Size of Vertical Transmission of GBS

The pooled effect size for vertical transmission of GBS was 51% (95% CI: 45, 58). The overall heterogeneity across the studies on vertical transmission of GBS from mother to child was moderate (I^2^ = 57.11%, *p* < 0.001).

### Publication Bias for Vertical Transmission of GBS

**Figure 5:**
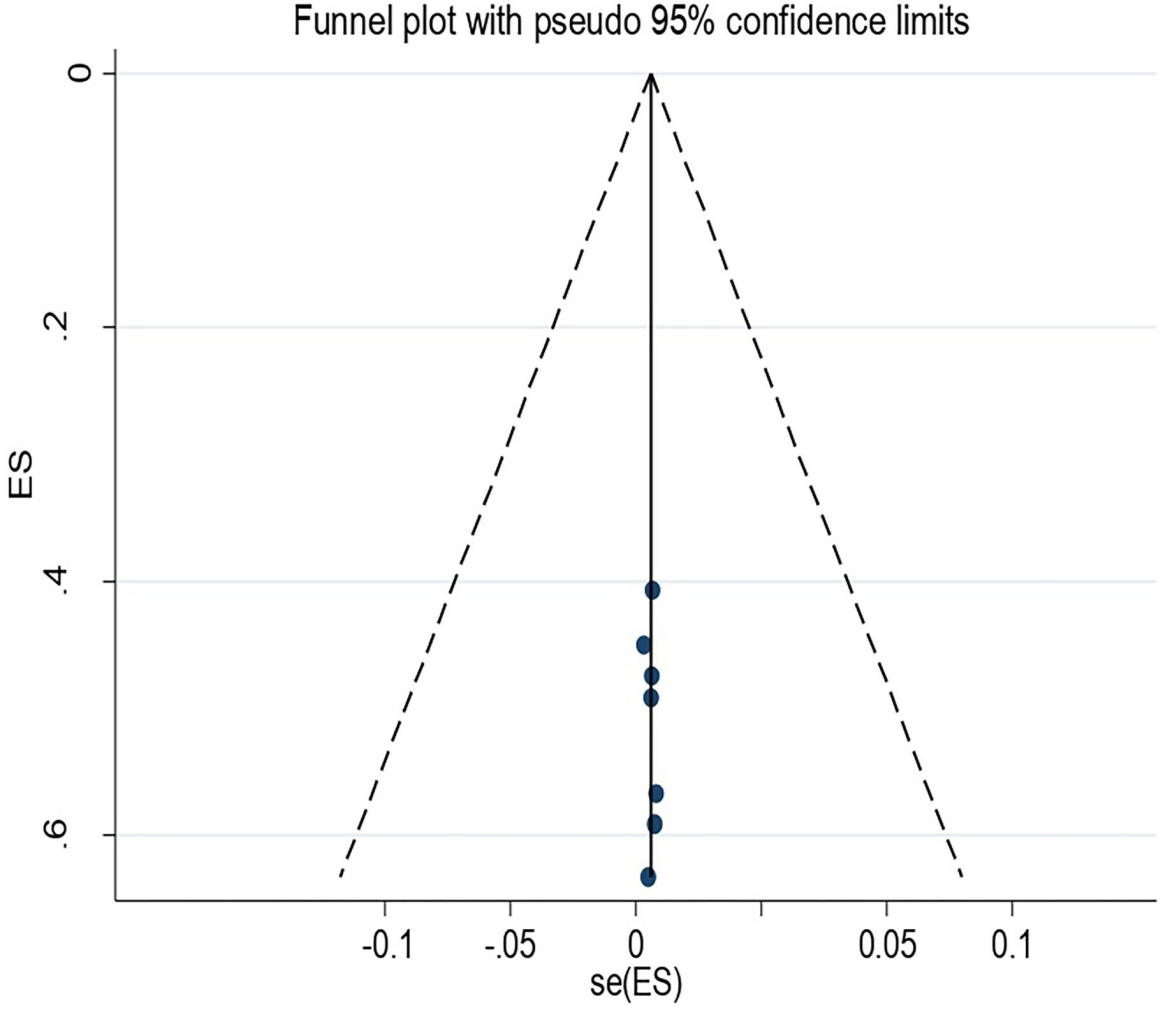
Funnel plot for vertical transmission of Group B streptococcus among pregnant women in Ethiopia 2022.

The Egger's regression test revealed that there was no evidence of publication bias among the included studies (*p* = 0.491). In addition, a visual inspection of the funnel plot also confirmed the absence of asymmetry (Figure 5).

### The Pooled Effect Size of Antibiotic Susceptibility of GBS

Among the 19 studies included in the analysis, 11 studies reported susceptibility of GBS to penicillin and erythromycin and 10 studies reported susceptibility of GBS to ampicillin and clindamycin. The highest pooled proportion of antibiotic susceptibility to GBS was observed in vancomycin, i.e., 99% (95% CI: 98, 100), followed by ampicillin, i.e., 94% (95% CI: 92, 97) (Table 2). Nonetheless, tetracycline is the lowest susceptible antibiotics, i.e., 23% (95% CI: 9, 36) (Table 3) (38).

**Table 3:** Pooled proportion of Antibiotic susceptibility of GBS isolated from pregnant women in Ethiopia since December 2012 to November 2021.

**Antibiotic tested for GBS**	No of studies (*n* = 18)^*^	**Antibiotic susceptibility 95% CI**	**I^**2**^ (%)**	***P*-Value**
Vancomycin	9	99 (98, 100)	77.49%	0.00
Ampicillin	10	94 (92, 97)	91.46%	0.00
Penicillin	11	91 (88, 94)	96.24%	0.00
Ciprofloxacin	4	84 (76, 92)	52.01%	0.00
Erythromycin	11	77 (68, 87)	85.18%	0.00
Chloramphenicol	5	76 (62, 89)	86.56%	0.00
Clindamycin	10	75 (65, 84)	84.02%	0.00
Ceftriaxone	7	71 (58, 854)	86.44%	0.00
Tetracycline	4	23 (9, 36)	85.27%	0.00

* *A study can be included in one or more categories; CI, confidence interval; GBS, group B streptococci*.

## Discussion

This systematic review and meta-analysis aimed at examining the recto-vaginal colonization of GBS, vertical transmission, and antibiotic susceptibility among pregnant women in Ethiopia. The pooled effect size for both recto-vaginal colonization of GBS and its vertical transmission to a newborn is alarming. The pooled estimate of the recto-vaginal colonization proportion of GBS was 15% (95% CI: 11, 19). Moreover, this study revealed that the pooled effect size for vertical transmission of GBS was 51% (95% CI: 45, 58).

The finding of the current meta-analysis is comparable with the meta-analysis conducted in Nicaragua, which had 14% overall estimates of maternal recto-vaginal GBS colonization proportion from 11 studies with 2,205 pregnant women (41). However, the finding from the current analysis is found to be low compared to the analysis of recto-vaginal colonization of GBS in Gabon, which was 29.8% (56). This discrepancy might be due to the variability in the number of studies involved in the meta-analysis, the variation in the number of pregnant women who participated in the two reviews, variations in the detection techniques (laboratory facilities) employed, and/or due to the biological factors among the study participants.

On the other hand, this review indicates that the pooled effect size for vertical transmission of GBS to neonates is 51%. This means that one out of two neonates, who is born from a mother with recto-vaginal colonization (RVC) of GBS, is at risk of being infected with GBS. This means that one in two pregnant women who were carriers of GBS had a chance of vertical transmission to their newborn. One of the possible explanations could be pregnant women who are infected and become carriers of GBS can transmit it to their babies easily. This might further be linked up with a delay in administering intrapartum antibiotics prophylaxis due to factors such as late presentation of the mother to the labor ward, precipitous delivery, or misidentification of risk factors, all of which can result in inadequate IAP cover (3, 33).

In a similar manner, the pooled finding of the current analysis indicated the presence of apprehension regarding the antibiotic susceptibility of GBS isolates from pregnant women. The isolates revealed susceptibility to vancomycin, ampicillin, as well as penicillin. This finding showed similarity with a meta-analysis conducted in Nicaragua, which confirmed GBS's high susceptibility to ampicillin and vancomycin (41, 57). However, in this study, the susceptibility is low for ciprofloxacillin, erythromycin, clindamycin, ceftriaxone, and tetracycline. The occurrence of the low susceptibility of GBS isolates might be due to the extensive use of antibiotics empirically for the treatment of different infectious diseases. It might also be due to the accessibility of these drugs without restriction in various areas with low cost and tradition of self-prescription.

Here, it should be noted that the expanded utilization of the beta-lactam antibiotics in the treatment of numerous infective clinical syndromes and the ease of accessibility of medication over the others also contributes to the emergence of a GBS with low susceptibility. Thus, utilization of clindamycin, erythromycin, and ceftriaxone should be directed by antimicrobial susceptibility testing. However, the absence of national guidelines on how to manage GBS colonization worsens its complication, both for pregnant women and newborns in Ethiopia. Furthermore, consistent with other studies, the non-susceptibility of GBS to tetracycline serves as a wake-up call to the need to discontinue the use of tetracycline for either treatment or prophylaxis.

Finally, in this review, a prolonged premature rupture of membrane showed association with recto-vaginal colonization of GBS. This is because GBS infection of the choriodecidua causes dysfunction of the cytokeratin network in the amniotic epithelium. Subsequently, it results in membrane weakening which leads to early rupture of the membrane (58). Thus, intrapartum antibiotic prophylaxis is essential in tackling such obstetric complications caused by GBS.

### Strengths and Limitations

The investigators used extensive and comprehensive search strategies from multiple databases. Published as well as unpublished studies and gray literature were included. Studies were evaluated for methodological quality using a standardized tool. Although the literature search was systematic and assessed all related studies within the desired scope, some relevant publications, e.g., publications reported in non-English language and local languages, may have been missed. Studies with abstracts were the only ones included. This could affect the finding's inclusiveness.

## Conclusion

In conclusion, nearly one out of seven pregnant women in Ethiopia had recto-vaginal colonization of GBS. As a result, half of the general pregnancy in Ethiopia is complicated by the vertical transmission of GBS. Vancomycin is a drug of choice for GBS in Ethiopia. Hence, the review highlights that the policies and programs should find mechanisms to address the reduction of GBS and vertical transmission. It is also recommended that revising first-line antibiotic treatment of GBS is vital to tackle its complications. In addition, it is better to consider the screening of pregnant women for GBS colonization.

## Data Availability Statement

The original contributions presented in the study are included in the article/Supplementary Material, further inquiries can be directed to the corresponding author.

## Author Contributions

HB, TG, AD, KS, and BB conceived and designed the review. HB, TG, MK, DT, and GT carried out the draft of the manuscript. HB is the PI of the review. HB, TG, AD, and KS developed the search strings. AE, TG, KS, DT, and BB screened and selected studies. HB, AE, MK, GT, SM, BE, and AD extracted the data and evaluated the quality of the studies. AE, KS, SH, AD, LR, DT, MK, YD, and AA carried out the analysis and interpretation. HB, TG, AE, DT, BB, AD, GT, MK, YD, AA, and KS rigorously reviewed the manuscript. All authors read and approved the final version of the manuscript.

## Conflict of Interest

The authors declare that the research was conducted in the absence of any commercial or financial relationships that could be construed as a potential conflict of interest.

## Publisher's Note

All claims expressed in this article are solely those of the authors and do not necessarily represent those of their affiliated organizations, or those of the publisher, the editors and the reviewers. Any product that may be evaluated in this article, or claim that may be made by its manufacturer, is not guaranteed or endorsed by the publisher.

## Abbreviations

MeSH: Medical Subject Headings
OR: Odds Ratio
PRISMA: Preferred Reporting Items for Systematic Reviews and Meta-analysis
GBS: Group B streptococcus
WHO: World Health Organization.
