# Supplementary material for: Prevalence of Group B Streptococcus Recto-Vaginal Colonization, Vertical Transmission, and Antibiotic Susceptibility Among Pregnant Women in Ethiopia: A Systematic Review and Meta-Analysis
Source: Front Public Health. 2022 May 16;10:851434. doi: 10.3389/fpubh.2022.851434 (PMC9149289; doi:10.3389/fpubh.2022.851434)
Supplement: Supplementary file 2 [file Table_2.DOCX]

***Additional file 2*; Sample search string for CINHAL database, EBSCOhost Interface**

| **#** | **Query** | **Limiters/Expanders** | **Last Run Via** | **Results** |
| --- | --- | --- | --- | --- |
| **S5** | **S1 AND S2 AND S3 AND**  **S4** | Expanders - Also search within the full text of the articles  Search modes - Boolean/Phrase | Interface - EBSCOhost Research Databases  Search Screen - Advanced Search  Database - CINAHL | **3** |
| S4 | Ethiopia | Expanders - Also search within the full text of the articles  Search modes - Boolean/Phrase | Interface - EBSCOhost Research Databases  Search Screen - Advanced Search  Database - CINAHL | **6578** |
| S3 | colonization OR Vertical  transmission OR  antimicrobial susceptibility | Expanders - Also search within the full text of the articles  Search modes - Boolean/Phrase | Interface - EBSCOhost Research Databases  Search Screen - Advanced Search  Database - CINAHL | 19,606 |
| S2 | group b streptococcus in  pregnancy | Expanders - Also search within the full text of the articles  Search modes - Boolean/Phrase | Interface - EBSCOhost Research Databases  Search Screen - Advanced Search  Database - CINAHL | 690 |
| S1 | Rectovaginal OR Vaginal OR Rectal | \|  \| Expanders - Also search within the full text of the articles  Search modes - Boolean/Phrase \| \| --- \| --- \| | Interface - EBSCOhost Research Databases  Search Screen - Advanced Search  Database - CINAHL | 45,778 |

End
