# Supplementary material for: Prevalence of Group B Streptococcus Recto-Vaginal Colonization, Vertical Transmission, and Antibiotic Susceptibility Among Pregnant Women in Ethiopia: A Systematic Review and Meta-Analysis
Source: Front Public Health. 2022 May 16;10:851434. doi: 10.3389/fpubh.2022.851434 (PMC9149289; doi:10.3389/fpubh.2022.851434)
Supplement: Supplementary file 3 [file Table_3.DOCX]

**Appraisal**

Table 1: Critical appraisal check list of quantitative studies of Group B Streptococcus Recto-vaginal Colonization, Vertical Transmission and Antibiotic Susceptibility among Pregnant Women in Ethiopia: (1 =yes, 0=no/not mentioned); total score=8

| Studies | Q1 | Q2 | Q3 | Q4 | Q5 | Q6 | Q7 | Q8 | Total score | Remark |
| --- | --- | --- | --- | --- | --- | --- | --- | --- | --- | --- |
| Ali et al | Y | Y | Y | Y | Y | Y | Y | Y | 8/8 |  |
| Woldu et al | N | Y | Y | Y | Y | Y | Y | Y | 7/8 | Inclusion criteria not well explained |
| Shiferaw etal | Y | Y | Y | Y | Y | Y | Y | Y | 8/8 |  |
| Yadeta et al | Y | Y | Y | Y | Y | Y | Y | Y | 8/8 |  |
| Ali et al | Y | Y | Y | Y | Y | Y | Y | Y | 8/8 |  |
| Mengist et al | Y | Y | Y | Y | Y | Y | Y | Y | 8/8 |  |
| Girma et al | Y | Y | Y | Y | Y | Y | Y | Y | 8/8 |  |
| Ali et al | Y | Y | Y | Y | Y | Y | Y | Y | 8/8 |  |
| Giachew et al | Y | Y | Y | Y | Y | Y | Y | Y | 8/8 |  |
| Schonfeld et al | Y | Y | Y | Y | U | Y | Y | Y | 8/8 |  |
| Mohamed et al | Y | Y | Y | Y | Y | Y | Y | Y | 7/8 |  |
| Mengist et al | Y | Y | Y | Y | Y | Y | Y | Y | 8/8 |  |
| Gebremeskel et al | Y | Y | Y | Y | N | Y | Y | Y | 7/8 | The way confounding controlled was not stated |
| Fantahun et al | Y | Y | Y | Y | Y | Y | Y | Y | 8/8 |  |
| Alemseged et al | Y | Y | Y | Y | Y | Y | Y | Y | 8/8 |  |
| Assefa et al | Y | Y | Y | Y | Y | Y | Y | Y | 8/8 |  |
| Gizachew et al | Y | Y | Y | Y | Y | Y | Y | Y | 8/8 |  |
| Yadeta et al | Y | Y | Y | Y | Y | Y | Y | Y | 8/8 |  |
| Leykun et al | Y | Y | Y | Y | Y | Y | Y | Y | 8/8 |  |

Notes:

Q1 - Were the criteria for inclusion in the sample clearly defined?

Q2 - Were the study subjects and the setting described in detail?

Q3 - Was the exposure measured in a valid and reliable way?

Q4 - Were objective, standard criteria used for measurement of the condition?

Q5 - Were confounding factors identified?

Q6 - Were strategies to deal with confounding factors stated?

Q7 - Were the outcomes measured in a valid and reliable way?

Q8 - Was appropriate statistical analysis used?

Abbreviations: Y, yes; N, no; U, unclear.
